# Value Addition to African Natural Product-Based Drug Discovery Initiatives

**DOI:** 10.1021/acs.jnatprod.5c00446

**Published:** 2025-07-29

**Authors:** Godfrey Mayoka, Peter Mubanga Cheuka, Phanankosi Moyo, Godwin Akpeko Dziwornu, Denzil Beukes

**Affiliations:** † Department of Drug Design and Optimization, Helmholtz Institute for Pharmaceutical Research Saarland, Helmholtz Centre for Infection Research, Campus E8.1, Saarbrucken 66123, Germany; ‡ School of Pharmacy, Jomo Kenyatta University of Agriculture and Technology, P.O. Box 62000, 00200 Nairobi, Kenya; § Department of Chemistry, School of Natural Sciences, 108234 University of Zambia, P.O Box 32379, 10101 Lusaka, Zambia; ∥ Biodiscovery Center, Department of Chemistry, Faculty of Natural and Agricultural Sciences, University of Pretoria, Natural Science 1 Building, Private Bag X20, Hatfield 0028, South Africa; ⊥ Drug Discovery and Development Centre, Department of Chemistry, 37716 University of Cape Town, 7701 Rondebosch, Cape Town, South Africa; # School of Pharmacy, University of the Western Cape, Robert Sobukwe Rd., Bellville, Cape Town 7535, South Africa

**Keywords:** African Natural Product, Value Addition, Drug Discovery Initiatives, Bioactivity

## Abstract

Natural products are vital to drug discovery, yet Africa’s vast biodiversity remains underutilized. This perspective examines barriers limiting Africa’s impact—such as weak infrastructure, limited translational capacity, and minimal integration of medicinal chemistry. We advocate for advancing beyond basic extraction to include systematic isolation, pharmacokinetics studies, and semisynthetic derivatization. Emphasis is placed on integrating AI, cheminformatics, and biotransformation, alongside embedding drug discovery training into academic curricula. Strengthening regional networks, fostering interdisciplinary collaborations, and securing Africa-sensitive funding are essential. Strategic implementation of these actions will enable Africa to harness its natural resources for global drug discovery and address local health challenges.

## Graphical abstract

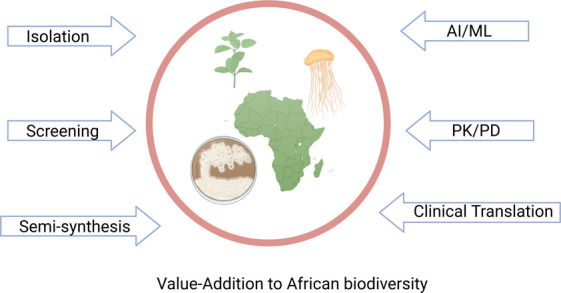

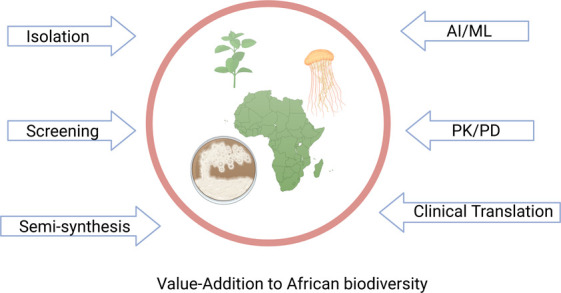

## 1 Introduction

The pivotal role of natural products in the drug discovery landscape is irrefutable. Historically, these products have served as the foundation for numerous therapeutics, either as sources of active pharmaceutical ingredients or as chemical templates for semisynthetic modifications. Their contributions span diverse therapeutic areas, underscoring their enduring significance in shaping modern medicine.^1,2^ Yet, despite their immense value, natural product-based drug discovery has gradually fallen out of favor with the pharmaceutical industry due to several reasons, including concerns about the structural complexity of natural compounds, the sustainability of raw material supplies, and inefficiencies associated with isolation, purification, and characterization processes.^3^ Furthermore, projections of return on investment have driven many large pharmaceutical companies to pivot toward small-molecule synthetic approaches, often deemed more predictable and cost-effective.^4^

Nonetheless, natural products remain a critical element in the search for new therapeutics, particularly in regions like Africa, where they play a central role in traditional medicine and are increasingly integrated into modern healthcare practices.^5^ The long-standing reliance on natural products across Africa is deeply rooted in folklore knowledge, accessibility, affordability, and perceived safety.^6^ Despite their widespread use, in the form of herbal and polyherbal products, the full potential of Africa’s natural resources remains largely untapped. As one of the most biodiverse regions in the world, Africa hosts a vast array of unique flora, fauna, and microorganisms.^7^ This unparalleled biodiversity positions the continent as a potential powerhouse for natural product-based drug discovery. Yet, much of this promise remains unrealized due to limited infrastructure, funding constraints, and gaps in scientific knowledge.

Currently, natural product-based medicine in Africa is largely confined to traditional uses with minimal integration into formal drug discovery pipelines. While these practices still address existing gaps in healthcare, especially in underserved and remote areas, they often fail to maximize the therapeutic potential of natural products. Concerns over toxicity, lack of standardization, and suboptimal pharmacological profiling frequently undermine their broader application and wider acceptance.^8^ Addressing these challenges demands a strategic shift to modernize and enhance the natural product drug discovery process in Africa, ensuring the continent’s unique resources are fully leveraged for both global and local health benefits.

Encouragingly, a few success stories have demonstrated the potential of African natural products in drug discovery. Compounds derived from plants and other natural sources have progressed through preclinical evaluation and even clinical application, providing tangible evidence of the untapped opportunities within this field. However, these achievements remain isolated, representing only a fraction of what could be accomplished with concerted and purposefully directed efforts. Transforming these sporadic successes into widespread and sustained progress requires Africa to adopt deliberate strategies to integrate natural products into mainstream drug discovery frameworks. This integration would not only harness the continent’s rich biodiversity but also position natural product research as a cornerstone of its scientific and healthcare ecosystems.

The challenges hindering African natural product-based drug discovery are multifaceted. Limited knowledge of pharmacological and pharmacokinetic profiles often prevents promising compounds from advancing to later stages of development. Infrastructural, funding, and methodological constraints not only inhibit the exploration of new chemical spaces but also deter researchers from pursuing careers in natural product-based drug discovery. These barriers underscore the urgent need for innovative and well-thought-out strategies, underpinned by responsive policy and regulatory frameworks, to unlock the full potential of Africa’s natural products.

We propose actionable strategies to address these challenges and realize the untapped potential of natural products in Africa. Key approaches proposed in this perspective include advancing beyond basic extraction and screening to isolate and characterize bioactive compounds, incorporating early pharmacokinetic profiling to optimize drug-like properties, and leveraging semisynthetic derivatization to enhance the efficacy and safety of promising compounds. In addition, emerging technologies, such as artificial intelligence and machine learning, are viewed as holding significant promise for accelerating target identification, mechanism-of-action studies, and predictive modeling, enabling more efficient drug discovery processes. Equally critical are deliberate collaborations and capacity-building initiatives, such as regional consortia to pool resources and expertise, fostering innovation while minimizing redundancy. Education and training programs tailored to natural product research are essential for developing a robust and talented human resource pipeline. Furthermore, integrating natural product research with medicinal chemistry and securing targeted funding for infrastructure development will be pivotal in ensuring broader and sustained progress in natural product-based drug discovery efforts. Addressing these challenges and implementing these strategies, can enable Africa to gain the impetus and more fully harness its natural resources, transitioning from a region reliant on external solutions to a global leader in natural product-based drug discovery. This transformation promises not only to advance global health but also to drive local socioeconomic development, aligning with the continent’s broader aspirations for self-reliance, driven by innovation.

## 2 Current Status of African Natural Product-Based Drug Discovery

### 2.1 African Perspective on Natural Product Drug Discovery

Natural products have long been the cornerstone of traditional medicine across Africa, serving as a primary source of therapeutics for centuries. In many cases, the use of natural products in modern times still follow, or borrow from, their prior traditional use in terms of preparation and utilization. While this may be resourceful in addressing current gaps in healthcare, concerns remain about empirical validation and ability to discern inferior or even harmful products.^8^ However, the landscape is slowly evolving with many African researchers becoming increasingly aware of the potential of nature-inspired bioactive compounds in modern drug discovery. Consequently, several African countries such as South Africa, Nigeria, Egypt, Kenya, and Ghana have made significant efforts in exploiting natural products research, supported by well-established research institutions investigating plant-based and microbial-derived compounds.

Notably, despite the continent’s rich biodiversity, African natural product research remains skewed toward plant-based sources, with microbial and marine bioactives receiving relatively less attention. A quantitative assessment using databases such as PubMed and Scopus reveal a disproportionately high number of studies on medicinal plants compared to microbial or marine natural products.^9^ This over-reliance on plant resources, while valuable, limits the exploration of potentially novel chemical scaffolds from underexplored sources such as endophytic fungi, extremophiles, and deep-sea microorganisms.

### 2.2 Promising African Natural Products

Several African-derived natural products and botanical medicines have demonstrated promising therapeutic potential, progressing from traditional use to preclinical and clinical research. The pursuit of botanical drug sources has been further inspired by the success of traditional medicine elsewhere, exemplified by the Chinese research leading to the discovery of artemisinin (Figure 1) from *Artemisia annua*.^10,11^ Artemisinin derivatives serve as highly effective antimalarial agents and crucial components of the first-line antimalarial drug combinations arsenal.^12^ While, to the best of our knowledge, no clinically relevant drugs have been discovered within Africa itself by African researchers, its flora and fauna have yielded valuable clinical agents, including physostigmine, ouabain, the vinca alkaloid vinblastine, and yohimbine, through research conducted internationally. (Figure 1).

**Figure 1:**
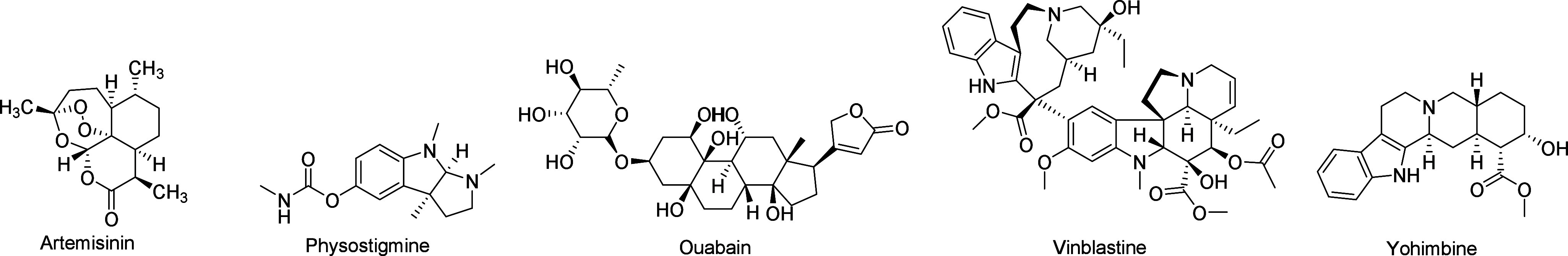
Structures of artemisinin and some natural product compounds isolated from African plants.

One of the earliest clinical discoveries originating from African folklore was the alkaloid physostigmine. This compound was first isolated from the calabar bean, *Physostigma venenosum*, used as an ordeal poison by the Efik people of Old Calabar in Nigeria.^13,14^ Scottish missionaries documented its use, prompting British scientists to investigate the plant leading to its subsequent isolation and naming.^14^ This molecule acts as an acetylcholinesterase inhibitor and is still clinically used to treat glaucoma, among other conditions. Notably, research elucidating its mechanism of action led to the discovery of acetylcholine and chemical neurotransmission, a contribution recognized with the 1936 Nobel Prize in Physiology or Medicine.^15^

Ouabain, a cardiac glycoside that inhibits the sodium/potassium (Na^+^/K^+^) ATPase pump, is found in *Strophanthus gratus*, a plant used in arrow poisons in East Africa.^16,17^ The name “ouabain” derives from the Somali word “waabaayo”. Léon-Albert Arnaud, a French chemist, first isolated ouabain from *S. gratus* seeds in 1882.^18^ In the early 20th century, ouabain was widely used globally for treating heart conditions. However, its clinical use has diminished over time due to the adoption of safer and longer-lasting cardiac drugs.

The vinca alkaloid vinblastine, a chemotherapeutic agent used against various cancers, targets microtubules, disrupting their formation and inhibiting cancer cell growth. Its discovery from the Madagascan plant *Vinca rosea* (now *Catharanthus roseus*) was serendipitous. Canadian scientists, Robert Noble and Charles Beer, at the University of Western Ontario initially evaluated *V. rosea* for its antidiabetic properties, based on its traditional use in Madagascar for diabetes treatment. While their experimental attempts showed no hypoglycemic effect in diabetic rats, they observed a profound effect on white blood cells. This prompted them to investigate the plant’s anticancer properties, leading to the isolation and discovery of vinblastine. Working with the pharmaceutical company Eli Lilly, they scaled up production for clinical trials and approval, establishing vinblastine as a foundational chemotherapeutic agent.^19–21^

Yohimbine, an indole alkaloid found in the tree *Pausinystalia yohimbe*, native to West and Central Africa, was traditionally used as an aphrodisiac.^22^ As an α_2_-adrenergic receptor antagonist, it was introduced into Western medicine as a treatment for erectile dysfunction.^23^ Its use has declined with the emergence of more effective agents, such as phosphodiesterase type 5 inhibitors like sildenafil (viagra). Despite this, yohimbine maintains a significant market share as a pharmaceutical agent and supplement. In 2023, the market value for yohimbine was estimated at US$83 billion,^24,25^ approximately 2.7% of Africa’s Gross Domestic Product (GDP) for that year. Interest in the herbal yohimbe also exists, though to a lesser extent than in yohimbine itself. Several other important bioactive compounds from Africa’s natural treasure trove have been identified through international collaborations exemplified by the efforts of the U.S. National Cancer Institute (NCI), as discussed later. Overall, the success of drugs isolated from African traditional plants validates their properties and the knowledge embedded in African folklore. Although these discoveries were made by researchers outside of Africa, the potential for further discoveries remains significant.

In addition to isolated compounds, several botanical medicines have gained commercial interest due to their therapeutic value. For example, extracts of *Prunus africana* are widely used in the treatment of benign prostatic hyperplasia,^26^ while *Hoodia gordonii* has attracted interest as an appetite suppressant with potential applications in obesity management.^27^

### 2.3 Challenges and Gaps in African Natural Product Drug Discovery

#### 2.3.1 Gaps in Mechanistic Understanding and Drug-like Properties

One of the greatest hurdles in African natural product drug discovery is the lack of in-depth mechanistic studies. As a result, many bioactive compounds exhibit promising *in vitro* activity, yet their molecular targets, signaling pathways, and mode of action remain poorly characterized. Without a comprehensive understanding of how these compounds exert their effects, their progression through the drug discovery pipeline is severely hindered. Devoid of understanding the mechanism of action raises concern over potential toxicity that may be unexplainable, hindering confidence in the pharmaceutical market.^6^ In addition, lacking the mechanistic information also denies the scientific community a rich resource in identifying potentially new pathways that could pave the way for novel therapies discovered using target-based approaches.

Similarly, a significant proportion of African natural products suffer from poor absorption, distribution, metabolism, excretion, and toxicity (ADMET) properties, limiting their potential as drug candidates. Many bioactive compounds have low aqueous solubility, which negatively affects their bioavailability due to limited transportation across biological barriers.^28,29^ Some of these demonstrate poor metabolic stability, leading to rapid degradation *in vivo*, while others demonstrate off-target effects, eliciting toxicity concerns.^28,29^

#### 2.3.2 Structural and Resource Constraints

Despite Africa’s immense potential in natural product drug discovery, critical infrastructure gaps continue to constrain progress. The lack of high-throughput screening (HTS) platforms significantly slows down compound evaluation efforts, while the limited availability of advanced analytical tools such as nuclear magnetic resonance (NMR) and mass spectrometry (MS) hampers timely and accurate structural elucidation. Moreover, the absence of dedicated medicinal chemistry expertise and synthesis laboratories across many institutions makes hit-to-lead optimization particularly difficult. Research efforts also remain fragmented, with minimal collaboration between universities, national research institutes, and industry, thereby limiting synergy and scalability.

In addition to infrastructure, funding continues to be a persistent bottleneck. Most African governments allocate less than 1% of GDP to scientific research.^30^ This chronic underfunding results in heavy dependence on international grants, which often come with restrictive conditions, including limitations on research scope, intellectual property, and partnership structures.

Another critical limitation is the frequent rediscovery of already known compounds, a common and costly challenge in African natural product research.^31^ This issue stems from limited access to updated chemical libraries and spectral databases, which impairs early dereplication and results in redundant findings rather than the identification of novel scaffolds. The over-reliance on conventional extraction techniques may also limit the chemical diversity of isolated metabolites. Compounding the problem is the general shortage of bioinformatics and cheminformatics expertise, which hinders systematic compound annotation and prioritization. Without modern digital tools and interdisciplinary integration, distinguishing new chemical entities from previously identified ones remains a major obstacle to innovation.

## 3 Value-Addition Strategies in African Natural Product Drug Discovery

### 3.1 Broadening Discovery Scope and Screening Approaches

Natural products have historically been a major source of pharmaceuticals, with plants and microorganisms yielding the highest number of drug candidates to date (Figure 2).^32^ More recently, marine organisms have emerged as a promising source for novel bioactive compounds (Figure 2).^33^ On the other hand, microorganisms have contributed the largest number of clinically approved natural product-based drugs or derivatives, with the *Streptomyces* genus being particularly prolific in producing antibacterial agents^34^ (Figure 2). While plants have played a lesser role in anti-infective drug discovery, they have been the primary source of antiparasitic, cardiovascular, and cancer therapeutics (Figure 2 and Supporting Information). Marine-derived compounds, on the other hand, have been especially successful in cancer drug development, with nearly 90% of marine-derived drugs falling within the oncology space (Figure 2 and Supporting Information).

**Figure 2:**
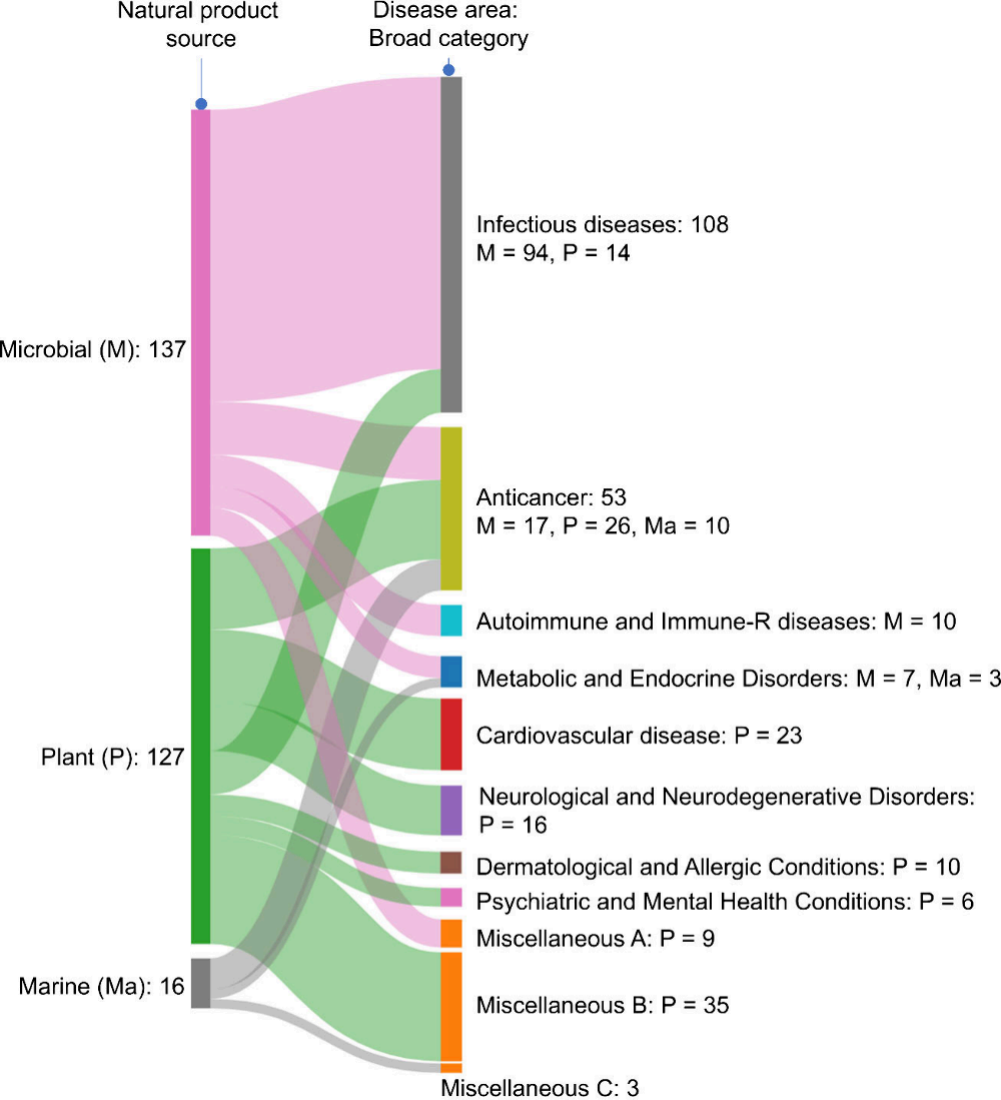
Proportion of clinically used drugs derived from microbial, plant, and marine sources. The Sankey diagram shows the extent of application of microbial-, plant-, and marine-derived drugs in different therapeutic areas. A, B, and C represent the proportion of miscellaneous disease areas in which microbial-, plant-, and marine-derived drugs find application. The figure has been developed using information retrieved from the following sources: refs 32 and 35–37 . See the Supporting Information for further details.

Despite Africa’s unmatched biodiversity, its natural product research has predominantly focused on plant-based drug discovery, often overlooking its vast microbial and marine ecosystems. Given the continent’s limited financial, human capital, and research infrastructure, researchers should strategically prioritize drug discovery in areas where African natural products have historically demonstrated success (as seen in Figure 2). While anti-infective research remains crucial, there is an urgent need to diversify research efforts into other therapeutic areas such as cancer, cardiovascular diseases, and neurological disorders. Additionally, harnessing African microbial and marine diversity could provide untapped opportunities for novel antibiotic and anticancer drug discovery. Indeed, broadening the scope of natural product research could unlock unique structural diversity and lead to the identification of first-in-class drugs useful, in not only addressing the continent’s pressing health challenges, but also contributing to the global drug discovery landscape.

Considering therapeutic indications, African natural product research has overwhelmingly focused on anti-infective agents, despite yielding limited success in developing novel antibiotics. While this remains an important area, narrow screening strategies risk overlooking compounds with potential applications in other therapeutic areas such as cancer, metabolic disorders, and neurodegenerative diseases. Broadening the scope of primary screening to include diverse disease targets could significantly enhance the utility of natural product-inspired scaffolds. To achieve this, strategic collaborations with institutions that offer high-throughput screening platforms for diverse biological targets can enhance efficiency in natural product drug discovery efforts. This would require establishing partnerships that provide access to specialized assays, such as cancer cell line screening, enzyme inhibition studies, and immune-modulatory assays, to increase the chances of identifying promising therapeutic leads beyond anti-infectives.

### 3.2 Biotransformation and Systematic Optimization of Natural Products

A substantial number of clinically approved drugs are derived from natural prodrugs—compounds that are pharmacologically inactive in their native state but become active upon biotransformation *in vivo*. Despite their therapeutic significance, such compounds are often overlooked during early screening because standard *in vitro* assays do not capture their metabolic activation. Recognizing and systematically incorporating biotransformation into natural product discovery workflows could greatly expand the pool of viable leads.

Several clinically established examples—such as romidepsin, salicin, digoxin, digitoxin, and codeine—underscore the potential of this approach (Figure 3). Their identification requires advanced biotransformation screening platforms that replicate metabolic pathways, including hepatocyte cultures, microsomal enzyme systems, and microbial bioconversion (e.g., with Alternaria species).^38^

**Figure 3:**
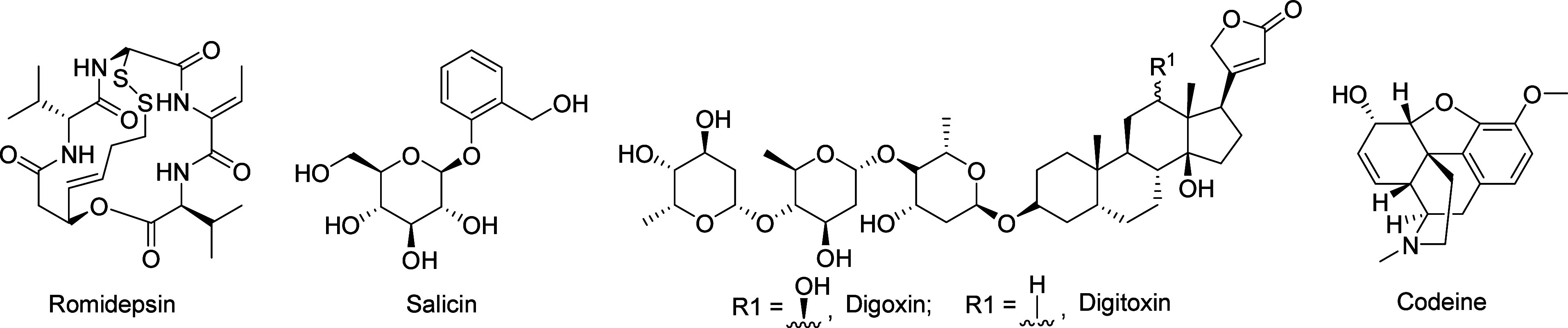
Structures of selected clinically relevant natural prodrugs.

In parallel, insights from traditional medicinal practices can offer valuable clues about transformation pathways. In some African cultures, burning medicinal plants forms part of therapeutic rituals—potentially triggering heat-induced chemical changes that enhance bioactivity. For instance, smoke from burning *Artemisia afra* demonstrated significantly superior antimicrobial activity compared to its solvent extracts.^39^ Such findings point to the untapped potential of thermal or environmental modifications as activation strategies. Figure 4 illustrates well-known prodrug conversions of salicin to salicylic acid and codeine to morphine, catalyzed by host enzymes.

**Figure 4:**
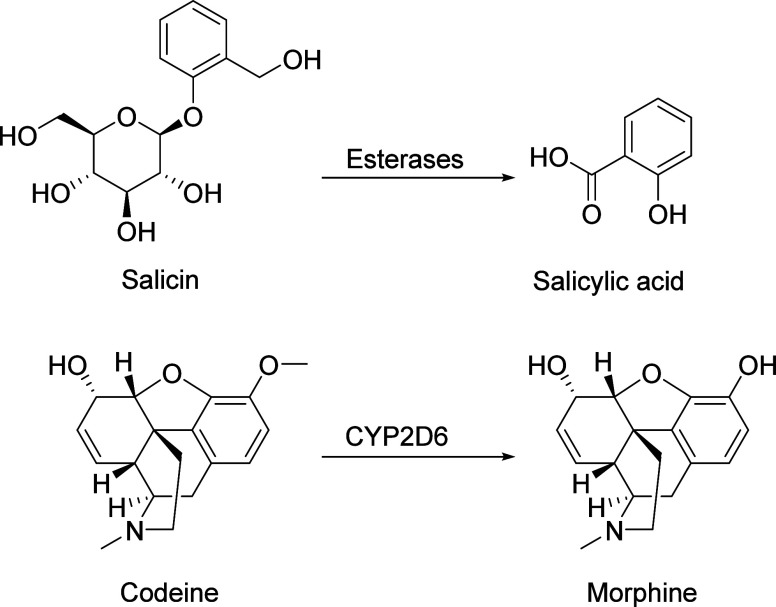
Prodrug conversion examples: (Top) salicin to salicylic acid via esterases; (Bottom) codeine to morphine via CYP2D6.

Despite such potential, many African natural product research efforts still stop at preliminary extraction and *in vitro* screening, without progressing to chemical, mechanistic, or pharmacokinetic characterization. This not only limits the translational value of findings but also contributes to the frequent rediscovery of known compounds—a challenge discussed earlier. Broader implementation of metabolomics and dereplication platforms such as Global Natural Product Social Molecular Networking (GNPS), SIRIUS, and Mzmine^40–42^ could vastly improve compound annotation and prioritization. However, adoption of these tools remains low across African institutions, partly due to limited access to high-resolution MS infrastructure and trained personnel.

Another underexplored dimension is the multicomponent nature of bioactive extracts, where synergistic or additive interactions may underlie observed activities. Yet, most studies focus solely on isolated *in vitro* end points, with limited evaluation of pharmacokinetic properties. This leaves many promising compounds without the preclinical data needed for downstream development.

To fully capitalize on Africa’s natural product potential, drug discovery pipelines must therefore integrate biotransformation screening, advanced dereplication technologies, and systematic pharmacokinetic profiling. These tools will be vital in identifying novel prodrugs, understanding compound synergy, and ensuring that leads possess the necessary bioavailability, metabolic stability, and safety profiles for further development.

### 3.3 Optimizing Bioactivity through Chemistry and Digital Innovation

Once bioactive compounds are isolated and characterized, medicinal chemistry can play a pivotal role in optimizing their drug-like properties. Semisynthetic derivatization—modifying the chemical structure of a natural compound to enhance its potency, efficacy, and pharmacokinetics—has been a widely successful strategy in several therapeutic areas. A notable example is artemisinin (Figure 1), where structural modifications of the parent molecule yielded derivatives such as artemether and artesunate, which are now the cornerstone of malaria treatment.

Conducting structural modifications to improve pharmacokinetic and pharmacodynamic profiles, as happens in mainstream small-molecule drug discovery projects, can help optimize primary hits derived from natural product drug discovery. In this regard, collaboration with medicinal chemists will be essential to address common challenges such as poor solubility, metabolic instability, and toxicity. Developing interdisciplinary research teams that integrate natural product chemists, pharmacologists, and computational biologists will ensure that promising bioactive compounds are effectively optimized for further preclinical development.

Artificial intelligence (AI) and machine learning (ML) have emerged as transformative tools in drug discovery, significantly accelerating target identification, mechanism-of-action studies, and predictive modeling for efficacy and toxicity.^43–45^ AI-driven approaches have already led to the development of small molecule drug candidates progressing into clinical trials.^46–49^ However, the natural products field appears to lag due to limited data sets, which are critical for training robust AI models.^50,51^ Despite the current challenges associated with applying AI/ML in natural product drug discovery, companies such as Enveda Biosciences^52,53^ are making significant strides in using AI-powered discovery models that accelerate drug candidate identification. To follow suit and benefit from current advances in the field, African research institutions must pool their limited computational resources, expertise, and data sets to develop AI-driven tools tailored to natural product research. Collaborations with AI-driven organizations such as Ersilia^54^ could be pivotal in building predictive models for compound activity, toxicity, and pharmacokinetics, significantly enhancing drug discovery efficiency.

### 3.4 Capacity Building, Strategic Partnerships, and Policy Support

Africa’s rich heritage in traditional medicine offers a strong foundation for natural product-based drug discovery. However, this potential remains underutilized due to gaps in specialized training, infrastructure, and policy frameworks. To harness this potential, a multifaceted approach encompassing education, collaboration, and regulatory support is essential.

#### 3.4.1 Education and Training

Integrating natural product chemistry, pharmacology, and medicinal chemistry into academic curricula across African institutions is crucial. Such integration will cultivate a skilled workforce capable of advancing drug discovery initiatives. Comprehensive training programs should encompass all stages of drug development, including compound isolation, structural modification, ADMET optimization, and clinical translation pathways. Establishing structured mentorship initiatives, collaborative research consortia, and clear career development trajectories will further nurture and retain talent within the continent.

#### 3.4.2 Strategic Collaborations and Funding

Africa’s geographical and genetic diversity provides a strong foundation for natural product drug discovery, yet research efforts remain fragmented. Establishing regional consortia that group research centers based on specialized expertise (e.g., marine natural products, microbial metabolites, and plant-derived drugs) can enhance productivity and prevent redundancy. Regular scientific forums, networking events, and collaborative platforms will facilitate knowledge exchange and promote strategic partnerships. Targeted funding for infrastructure development, research capacity-building, and technology acquisition is critical. Regional and international funding agencies should prioritize investment in high-throughput screening facilities, cheminformatics platforms, and medicinal chemistry laboratories to support long-term natural product research sustainability.

#### 3.4.3 Industry Engagement and Policy Frameworks

Collaborations with biotechnology companies can significantly accelerate the translation of promising natural product discoveries from academia into commercial or clinical applications. Partnerships with biotech firms provide access to advanced research facilities, technical expertise, and financial support, crucial for progressing drug candidates through clinical development phases. Establishing an African pharmacopoeia—a standardized reference for the efficacy, safety, and sourcing of African natural products—would enhance credibility and facilitate integration into global drug discovery pipelines. Policymakers should also consider implementing incentives that support innovation, streamline approval processes, and foster collaborations between academia and industry.

Through addressing educational gaps, fostering strategic partnerships, and strengthening policy support, Africa can unlock its immense potential in natural product-based drug discovery, contributing significantly to global health advancements.

## 4 Case Studies and Successful Models of Nature-Based Drug Discovery

### 4.1 The NCI Natural Products Repository

The U.S. NCI stands as one of the most explicit examples of the capacity and impact of international, cross-continental collaboration in natural product-based drug discovery.^55^ The NCI maintains one of the world’s most expansive natural products repositories, encompassing an extensive array of biological specimens collected from across the globe—including a significant number from Africa. Through long-standing collaborations with institutions such as the Missouri Botanical Garden and the University of Illinois at Chicago, more than 80,000 plant samples have been collected from the African continent and its surrounding regions. This is in addition to approximately 20,000 marine invertebrates and algae and over 16,000 microbial strains.

Building on this rich repository, the NCI launched the Program for Natural Product Discovery, which established a high-throughput, prefractionated extract library aimed at overcoming long-standing challenges associated with screening crude natural extracts.^32^ This initiative includes over 125,000 extracts processed through standardized extraction methods and separated into more than one million fractions using high-throughput solid-phase extraction (SPE) platforms. Many of these prefractionated extracts were derived from plants, marine organisms, and microbial cultures sourced from biodiversity hotspots in Ghana, Madagascar, Nigeria, Kenya, and South Africa.

The monumental achievements of the NCI’s African biodiversity work are reflected in the isolation of numerous bioactive compounds, several of which have advanced through preclinical and clinical development milestones.^56,57^

These collections are not only essential for diversifying the global natural product scaffold library but also represent a valuable archive of Africa’s rich chemical biodiversity. They provide opportunities for African scientists to engage in downstream bioassays, structural elucidation, and compound optimization. Importantly, the NCI’s collaborative efforts involved African partners in sample collection and research processes, fostering knowledge sharing and skills transfer, particularly in advanced analytical and discovery methodologies.

Clearly, while relatively few clinical drug candidates have so far originated from African research institutions, African biodiversity has made significant contributions to the global drug discovery pipeline. Increasing the visibility and involvement of African scientists in such international efforts would strengthen local research capacity, ensure equitable benefit-sharing, and promote African-led innovation. This calls for strategic partnerships focused on repatriating data, promoting coauthorship, and strengthening institutional capabilities.

However, as with many international initiatives, the continuity and sustainability of such collaborations often depend on the geopolitical and funding priorities of the lead country. Promising programs risk disruption or termination when national or international policy directions shift unfavorably.

Moving forward, aligning African research institutions—and by extension, national governments—with global repositories and screening initiatives such as those of the NCI will be crucial. Active participation in funding, data generation, and open-access collaborations could represent a transformative step toward meaningful inclusion in the global drug discovery ecosystem. It would also reaffirm Africa’s strategic value and relevance in natural product innovation.

### 4.2 Creation of African Natural Product Databases

Several African-led initiatives have significantly advanced natural products research, particularly through the development of natural product databases. These databases have played a crucial role in cataloging bioactive compounds, providing researchers with access to valuable chemical and biological data.

The Northern African Natural Products Database (NANPDB), first disclosed in 2017,^58^ comprises approximately 4,500 natural product compounds isolated from diverse sources, including plants, endophytes, animals, fungi, and bacteria. It is the most extensive collection of annotated compounds derived from organisms native to Northern Africa, offering insights into their physicochemical properties and predicted toxicity.

Expanding on this initiative, the African Natural Products Database (ANPDB) provides a more comprehensive collection of slightly over 5000 natural products derived from Northern and Eastern African regions.^59^ As the largest repository of natural products extracted from native African organisms—including plants, microorganisms, animals, and marine life—ANPDB offers detailed information on compound names, chemical structures, source organisms, references, biological activities, and, where available, modes of action. Its searchable interface allows users to retrieve data using various criteria such as compound names, chemical structures, source organisms, and keywords. Additionally, the database features a region-specific data access function and enables researchers to download chemical structures for virtual screening experiments, thereby facilitating computational drug discovery.

Recognizing the absence of a dedicated natural product database for South African compounds, researchers in South Africa established the South African Natural Compound Database (SANCDB) in 2015.^60^ Initially containing 600 natural product compounds extracted from journal articles, book chapters, and theses, the database was later updated and in 2021, it housed slightly over 1000 compounds.^61^ The SANCDB features a user-friendly web interface, allowing researchers to search compounds by various parameters. Each compound page includes links to original referenced work, ensuring full traceability. Furthermore, the database includes a submission pipeline, enabling researchers to contribute newly identified compounds, fostering a dynamic and continuously expanding resource.

In 2014, another natural product library, ConMedNP, was reported, containing approximately 3,200 natural product compounds isolated from Central African plants.^62^ This database includes calculated physicochemical parameters that serve as predictors of oral bioavailability based on Lipinski’s Rule of Five. Comparative analysis with other libraries demonstrated that ConMedNP contained the largest collection of three-dimensional drug-like, lead-like, and fragment-like natural products from the Central African forests. Prior to its public disclosure, AfroDb, a similar library containing compounds from medicinal plants across the African continent, was introduced in 2013.^63^ Like ConMedNP, the compounds in AfroDb have been evaluated for their drug-like, lead-like, and fragment-like properties, making them valuable resources for pharmaceutical development.

While considerable progress has been made in establishing virtual libraries cataloging African natural products, a critical gap remains: the absence of centralized physical libraries containing these compounds. At present, natural product samples are scattered across multiple laboratories in Africa, limiting accessibility for large-scale drug discovery efforts. The full potential of Africa’s natural product wealth can only be realized if these compounds are physically stored in centralized repositories, ensuring easy access for HTS programs aimed at hit identification and lead optimization. Alternatively, a well coordinated, decentralized network of regional repositories—with clearly documented locations and structured accessibility protocols—could serve as an effective alternative, enabling researchers to request or purchase compounds for HTS-driven drug discovery initiatives.

### 4.3 Intra-Africa Consortia

Beyond the establishment of virtual libraries, efforts have also been directed toward forming consortia that leverage diverse regional capacities to advance natural-product-based drug discovery. These initiatives aim to foster collaborations, enhance knowledge exchange, and provide essential research infrastructure across Africa.

One such initiative is the Natural Products Research Network for East and Central Africa (NAPRECA),^64^ a consortium of natural product researchers spanning East and Central Africa. NAPRECA champions the discovery of natural products for applications in human and animal health as well as agrochemicals. With branches in Botswana, Cameroon, the Democratic Republic of Congo, Egypt, Ethiopia, Kenya, Madagascar, Rwanda, Sudan, Tanzania, Uganda, and Zimbabwe, the network plays a pivotal role in research capacity building. It runs postgraduate scholarships in natural product science and facilitates networking and knowledge exchange through biannual symposia and thematic workshops. Since 1988, it has benefited from support by the International Science Programme (ISP), helping sustain its activities and outreach.

The African Research Network (ARN) of the Society for Medicinal Plants and Natural Products Research is another initiative that promotes intra-African and international collaborations, knowledge sharing, and capacity building in the field of natural products research. ARN has organized various scientific events, including workshops, webinars, and symposia, to strengthen research linkages and advance the field.^65^

The Network for Analytical and Bioassay Services (NABSA) has also contributed significantly to the development of natural products research in Africa.^66^ NABSA comprises laboratories housed within the Departments of Chemistry at Addis Ababa University (Ethiopia), the University of Nairobi (Kenya), and the University of Botswana (Botswana). Encouraged by the International Organization for Chemistry in Development (IOCD), NABSA has facilitated short-term research visits, allowing visiting researchers to conduct analytical and preparative experiments, involving high-performance liquid chromatography (HPLC), NMR, and MS. The network has also enabled scientists to ship samples for off-site analysis, increasing accessibility to specialized analytical services. However, the current operational status of NABSA remains unclear.

In West Africa, the West African Network of Natural Products Research Scientists (WANNPRES) has championed natural products research since its inception in 2002.^67^ The network comprises scientists from 15 West African countries, including Ghana, Togo, Benin Republic, Côte d’Ivoire, Mali, Senegal, Burkina Faso, Cameroon, Niger, Mauritania, Chad, Gambia, Liberia, Sierra Leone, and Nigeria. WANNPRES is founded on five key pillars: promoting natural products research, fostering intra-African and international linkages, championing biodiversity conservation and sustainable use, and disseminating research findings. Despite the absence of a consistent funding stream, WANNPRES has received financial support for scientific meetings from organizations such as the International Foundation for Science (IFS), the WHO Regional Office for Africa, the Organization for the Prohibition of Chemical Weapons (OPCW), and The Academy of Sciences for the Developing World (TWAS).

While multiple African initiatives continue to drive research and development into natural products, sustaining their activities remains a major challenge.^66,68^ A constrained funding pool is a persistent issue, as many of these networks heavily depend on external funding from foreign organizations, which is often time-bound. Once funding cycles end, many networks struggle to sustain their operations, leading to their eventual collapse. Those that continue to thrive, often benefit from consistent foreign funding streams or maintain affiliations with international organizations, allowing them to maintain their activities despite financial constraints.

Ensuring the long-term sustainability of these networks will require more diversified funding models, stronger local institutional support, and the development of innovative partnerships that prioritize Africa-led research autonomy. Addressing these challenges is crucial for enabling African scientists to fully harness the continent’s natural product potential for impactful drug discovery and development.

### 4.4 Learnings from Global Success Stories

Natural product-inspired drug discovery has demonstrated remarkable success globally, offering valuable lessons for Africa’s natural product-based drug discovery initiatives. Several countries have successfully integrated traditional medicine into mainstream healthcare, ensuring both scientific validation and widespread clinical application of herbal medicines.

One of the most prominent examples is China, where traditional Chinese medicine (TCM) is integrated into the healthcare system alongside conventional medicine.^69^ TCM has played a crucial role in drug discovery, leading to the identification of bioactive natural products that continue to be integral to modern pharmaceutical care. Among these, artemisinin, a potent antimalarial compound, remains one of the most celebrated successes. Artemisinin, isolated from the plant *Artemisia annua*, was historically documented for its medicinal properties in the ancient Chinese medical text A Handbook of Prescriptions for Emergencies.^70^ This discovery, pioneered by Youyou Tu, has saved millions of lives in malaria-endemic regions, particularly in Africa, through its inclusion in artemisinin-based combination therapies (ACTs). Tu’s groundbreaking work earned her the 2015 Nobel Prize in Physiology or Medicine, highlighting the significance of traditional knowledge in modern drug discovery. China has also invested heavily in clinical trials to scientifically validate traditional Chinese medicinal herbs, ensuring rigorous assessment of their safety and efficacy.^71^ This approach, which combines clinical evaluation with research to identify and understand active ingredients, has significantly facilitated quality control and standardization of herbal medicines.

Beyond China, India’s Ayurveda-based drug discovery provides another successful model. India has integrated Ayurvedic medicine into its healthcare framework, with government-backed institutions such as the Central Council for Research in Ayurvedic Sciences (CCRAS) driving the scientific exploration of medicinal plants.^72^ Notable drugs, such as reduced-risk antidiabetic formulations derived from *Gymnema sylvestre* and *Tinospora cordifolia*, have emerged from scientifically validated Ayurvedic knowledge.^73,74^ The Indian government has also invested in bioprospecting, clinical validation, and standardization of Ayurvedic formulations, ensuring that traditional medicines meet international pharmaceutical standards.^75^

Japan has successfully modernized Kampo medicine, a system of traditional herbal medicine adapted from China, by incorporating it into mainstream medical practice.^76^ The Japanese Ministry of Health, Labour and Welfare oversees the regulation and standardization of Kampo medicines, many of which are now covered by the national health insurance system. Kampo formulations such as Sho-saiko-to (a liver-protective herbal blend used in managing hepatitis and liver fibrosis) have undergone clinical and pharmacological evaluations, leading to their acceptance in conventional medicine.^77^ The success of Kampo medicine underscores the importance of combining traditional knowledge with rigorous scientific validation to ensure safety, efficacy, and widespread adoption.

South Korea is yet another example worth pointing to, where the Korean Oriental Medicine Promotion Act has facilitated the integration of traditional Korean medicine (TKM) into national healthcare. South Korea has established research institutes dedicated to traditional medicine and promotes evidence-based studies on herbal formulations. As a result, ginseng-based therapies, widely used in immunomodulation and cognitive enhancement, have gained global recognition due to their scientifically validated benefits.^78,79^

These global success stories illustrate how Africa could leverage its extensive biodiversity and traditional medicinal knowledge to advance its natural product-based drug discovery. By establishing structured research frameworks, investing in clinical validation, and integrating traditional medicine into national health policies, Africa can position itself as a leader in natural product-based pharmaceutical development. The combination of scientific rigor, regulatory oversight, and traditional wisdom has proven effective in other regions and could serve as a blueprint for Africa’s future in drug discovery and development.

## 5 Conclusion

Africa is at the crossroads of immense opportunity and pressing challenges in natural product-based drug discovery. Despite its vast biodiversity and rich traditional medicine heritage, the continent has yet to fully translate these resources into a structured and sustainable pharmaceutical innovation ecosystem. While several initiatives, such as the creation of virtual libraries and intra-African research consortia, have laid the foundation for progress, much remains to be done to bridge the gaps in infrastructure, funding, standardization, and translational research.

Global success stories illustrate the transformative potential of integrating traditional medicine with modern scientific frameworks. China, India, Japan, and South Korea have demonstrated that structured policies, clinical validation, and regulatory oversight can propel natural products from folklore to mainstream healthcare. Africa can harness these lessons by implementing comprehensive research frameworks, promoting interdisciplinary collaboration, and establishing regulatory pathways that facilitate the transition from traditional medicine to validated pharmaceuticals.

The future of Africa’s natural product research hinges on adopting a multifaceted approach. Expanding research beyond plant-based sources to explore microbial, marine, and extremophile-derived compounds will diversify the chemical space available for drug discovery. Strengthening methodologies through early pharmacokinetic profiling, cheminformatics, and AI-driven analytics will optimize the discovery pipeline, making it more efficient and competitive. Building capacity through education, targeted funding, and collaborative networks will ensure sustained progress and the retention of talent within the continent.

It is only by shifting from fragmented efforts to coordinated and well-supported initiatives that Africa can position itself as a global leader in natural product-based drug discovery. This transition holds the promise of addressing both local and global health challenges while fostering economic growth, technological advancement, and self-reliance in pharmaceutical innovation. Through applying the right strategies, Africa’s unparalleled biodiversity can move beyond the current underutilized status to becoming a cornerstone of next-generation therapeutics and a beacon of scientific excellence on the global stage.

## Supplementary Material



## Notes

Additional methodology details for the construction of the Figure 2 Sankey diagram on the proportion of clinically used drugs derived from microbial, plant, and marine sources (XLSX)
